# Successful Shortening of Tuberculosis Treatment Using Adjuvant Host-Directed Therapy with FDA-Approved Phosphodiesterase Inhibitors in the Mouse Model

**DOI:** 10.1371/journal.pone.0030749

**Published:** 2012-02-03

**Authors:** Mamoudou Maiga, Nisheeth Agarwal, Nicole C. Ammerman, Radhika Gupta, Haidan Guo, Marama C. Maiga, Shichun Lun, William R. Bishai

**Affiliations:** 1 Center for Tuberculosis Research, School of Medicine, Johns Hopkins University, Baltimore, Maryland, United States of America; 2 Vaccine and Infectious Disease Research Center, Translational Health Science and Technology Institute, Gurgaon, India; 3 KwaZulu-Natal Research Institute for Tuberculosis and HIV (K-RITH), Durban, South Africa; 4 Howard Hughes Medical Institute, Chevy Chase, Maryland, United States of America; University of Maryland, United States of America

## Abstract

Global control of tuberculosis (TB), an infectious disease that claims nearly 2 million lives annually, is hindered by the long duration of chemotherapy required for curative treatment. Lack of adherence to this intense treatment regimen leads to poor patient outcomes, development of new or additional drug resistance, and continued spread of *M.tb.* within communities. Hence, shortening the duration of TB therapy could increase drug adherence and cure in TB patients. Here, we report that addition of the United Stated Food and Drug Administration-approved phosphodiesterase inhibitors (PDE-Is) cilostazol and sildenafil to the standard TB treatment regimen reduces tissue pathology, leads to faster bacterial clearance and shortens the time to lung sterilization by one month, compared to standard treatment alone, in a murine model of TB. Our data suggest that these PDE-Is could be repurposed for use as adjunctive drugs to shorten TB treatment in humans.

## Introduction

Tuberculosis (TB) is one of the oldest infectious diseases known to man, and its causative agent, the bacillus *Mycobacterium tuberculosis* (*M.tb.*) continues to plague the human population in the twenty-first century. The most recent World Health Organization (WHO) estimates of the global burden of TB are staggering: in 2009, there were 9.4 million incident and 14 million prevalent cases of active TB, with 1.6 million deaths attributable to this disease [1]. Even more alarming is the announcement by the WHO that almost 300,000 of these TB cases were associated with multi- and extensively drug resistant (MDR and XDR, respectively) strains of *M.tb.* TB takes this global toll despite the availability of effective chemotherapy regimens, which in part is due to the extensive length of treatment necessary for cure: six to nine months of continuous drug administration. Incomplete adherence to treatment regimens sets the stage for their failure, contributing both to the continued spread of *M.tb.* throughout communities, as well as to the development of drug-resistant bacilli. Thus, development of a curative TB chemotherapy regimen of shorter duration could have an enormous positive impact on TB control around the globe.

TB disease is characterized by an excessive immune response by the host, leading to severe tissue destruction in the lungs. This destructive process is necessary to ensure bacterial spread, as penetration into damaged airways allows for aerosol transmission of the bacilli. It is becoming increasingly evident that the immune pathology observed in TB patients is not solely an inappropriate host response, but rather represents a precarious balance of both host- and bacteria-induced signaling events [2], [3]. Drugs that manipulate this host-pathogen relationship via modulation of the host response to *M.tb.* infection thus represent a large resource of adjunctive therapy options that could help to improve bacterial clearance in TB patients.

The nucleotide 3′, 5′-cyclic adenosine monophosphate (cAMP) is a highly conserved second messenger signaling molecule involved in the regulation of many cellular processes, both for eukaryotic and prokaryotic systems [4]–[6]. The concentration of cAMP within a cell is determined by the activity of two types of enzymes: adenylate cyclases, which synthesize cAMP, and phosphodiesterases (PDEs), which break down the cyclic nucleotide [7]. Recent work in our laboratory has demonstrated that *M.tb.* can directly manipulate cAMP signaling pathways within infected host macrophages, and that this mycobacterial subversion of eukaryotic cAMP signaling influences bacterial survival in mice [2]. Hence, the cAMP-mediated signaling pathways of the host cell represent a network which, during an *M.tb.* infection, could be targeted with modulatory drugs to reduce bacterial survival.

In mammals, up to 11 classes of PDEs have been identified based on sequence and structure similarity, substrate specificity and N-terminal regulatory domain components, and pharmacologic inhibitors are available for PDE types I–V [8]. PDE types I–III can hydrolyze both cAMP and cyclic guanosine monophosphate (cGMP), but with variable efficiency, while type IV and type V PDEs specifically hydrolyze cAMP and cGMP, respectively. In addition to differing substrate specificities, each PDE type has unique expression and localization profiles. For example, type III PDEs are prominent in macrophages, endothelial cells, platelets and airway smooth muscle cells, while type V PDEs are expressed in the vascular smooth muscle of pulmonary arteries and veins and bronchial blood vessels, as well as in airway smooth muscle cells [9]. PDE inhibitors (PDE-Is) can thus be utilized as tools to investigate the role of these PDEs during *M.tb.* infection.

In this study, we examined the influence of PDEs in mouse model systems of TB. Specifically, we evaluated the impact of two Food & Drug Administration- (FDA-) approved PDE i(PDE-Is, cilostazol (a type III PDE-I) and sildenafil (a type V PDE-I), on bacterial survival and disease pathology in murine TB models. Our data indicate that administration of PDE-Is to *M.tb.*-infected mice results in decreased bacterial burden and overall pathology, and that the addition of PDE-Is to the standard treatment regimen shortens the time to bacterial clearance. Our data suggest that the repurposing of existing FDA-approved PDE-Is for adjunctive TB chemotherapy could shorten treatment duration in TB patients, which would significantly impact in the global struggle against TB.

## Results

### Evaluation of PDE-Is during *M.tb.* infection of monocytic cells

**Figure 1:**
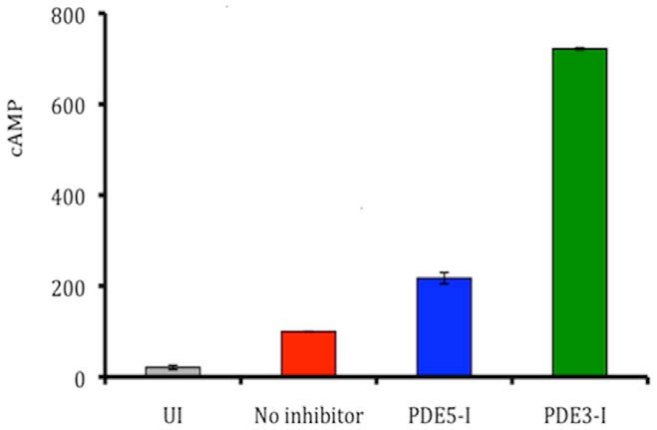
Intracellular cAMP levels increase within *M.tb.*-infected THP-1 human monocytic cells upon exposure to PDE-Is. The treated infected cells received 100 uM of PDE inhibitors (PDE-I 3, and 5 class) for 2 h followed by infection. UI: uninfected cells; the PDE5-I was 4-{[3′,4′-(Methylenedioxy)benzyl]amino}-6-methoxyquinazoline (MBM); and the PDE3-I was trequinsin. Results shown (mean and SD) represent two biological replicates, each with 2 technical replicates.

Previous work in our laboratory and by others has shown that mycobacterial infection of macrophages increases intracellular cAMP [2], [10], [11]. In an attempt to modulate intramacrophage cAMP levels, we administered PDE-Is to *M.tb.*-infected THP-1 human macrophage-like cells and subsequently measured intracellular cAMP concentrations. *M.tb.* infection produced approximately a 4-fold burst in intramacrophage cAMP levels, compared to uninfected cells (Fig. 1). Exposure of the infected cells to 100 µM of the type V PDE-I (PDE5-I) MBM (4-{[3′,4′-(Methylenedioxy)benzyl]amino}-6-methoxyquinazoline) increased the intracellular cAMP concentration by an additional 2.2-fold (p<0.0001, Friedman test), and exposure of the cells to the type III PDE-I (PDE3-I) trequinsin resulted in a nearly 8-fold additional increase in intracellular cAMP levels (p<0.0001, Friedman test). These results indicated that PDE3- and PDE5-Is could modulate the intramacrophage cAMP dynamic during *M.tb.* infection. MBM and trequinsin are cell biology reagents limited for use in the laboratory; however, the PDE3-I cilostazol and the PDE5-I sildenafil are FDA-approved drugs currently used primarily for the treatment of intermittent claudication and erectile dysfunction, respectively. Thus, we utilized these clinically relevant drugs to investigate the disease-modulating effects of PDE-Is in murine models of TB.

### Analysis of PDE-I monotherapy in *M.tb.*-infected BALB/c mice

**Figure 2:**
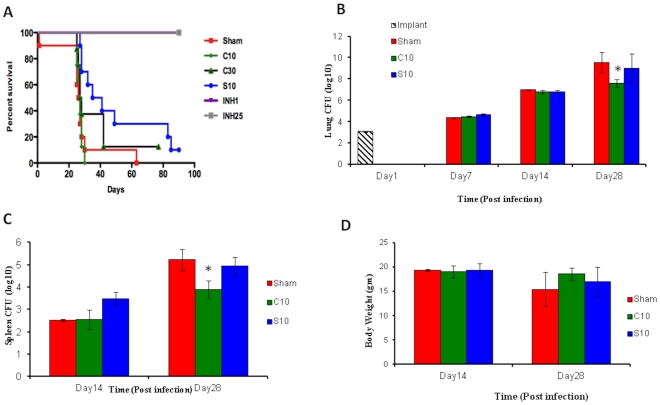
Administration of PDE-I monotherapy to *M.tb.*-infected mice shows therapeutic benefits by several parameters. (**A**) Time-to-death analysis; BALB/c mice infected with 3.6 log_10_CFU *M.tb.* were treated daily by oral gavage (starting the day after infection) with 10 or 30 mg/kg of cilostazol (C) or sildenafil (S). Isoniazid at 1 or 25 mg/kg (INH1 or INH25 respectively) and sham (PBS) were used as positive and negative controls, respectively. (**B–D**) BALB/c mice infected with 3.1 log_10_CFU *M.tb.* were treated daily by oral gavage (starting the day after infection) with 10 mg/kg cilostazol (C10), sildenafil (S10) or sham. At day 28 post-infection, lung CFU counts (**B**), spleen CFU counts (**C**) and body weight (**D**) were determined.

To evaluate if administration of cilostazol or sildenafil altered the course of TB in mice, we performed a time-to-death experiment. Six groups of 10 BALB/c mice were infected by aerosol with a relatively high dose of *M.tb.* (3.6 log_10_ colony forming units [CFU] implanted, as determined day 1 post-infection). In this model system, mice are infected with a relatively high inoculum (>3.5 log_10_CFU), which will cause untreated mice to die within the first month, allowing for the observation of differences in time-to-death. Drug administration was initiated the day after infection with the treatment groups as follows: cilostazol at 10 mg/kg (C10) and 30 mg/kg (C30); sildenafil at 10 mg/kg (S10); isoniazid (INH) at 1 mg/kg (INH1) and 25 mg/kg (INH25) as positive controls, and phosphate buffered saline (sham) as the negative control. The drugs were administered daily (5 days/week) by oral gavage for 45 days, and the time to death (post-infection) of each mouse was recorded. As expected, most of the sham-treated mice died within 30 days, and none of the INH-treated mice died during the experiment (Fig. 2, A). Mice receiving C10 experienced similar time-to-death as the sham-treated mice, while the S10-treated mice experienced increased survival relative to the sham-treated mice (Kaplan-Meier p values of 0.04 and 0.005, respectively). Thus, these data suggested that treatment with PDE-Is could increase mice survival following high-dose aerosol infection with *M.tb.*

To examine the impact of PDE-I administration on bacterial burden and overall pathology, BALB/c mice were infected with *M.tb.* via the aerosol route resulting in an implantation of 3.1 log_10_ colony forming units (CFUs) in the lungs (determined on day 1 post-infection). In this model system, mice are infected with a lower inoculum than in the time-to-death model (<3.5 log_10_CFU) in order for the sham-treated mice to survive throughout the length of the experiment, allowing for CFU comparisons to this critical control group. PDE-I administration was initiated on the first day post-infection. S10 and C10, as well as a saline control (sham) were administered daily (5 days/week) by oral gavage. During the first two weeks of treatment, mice receiving either of the PDE-Is did not exhibit different lung or spleen CFU counts than the sham-treated mice (Fig. 2, B and C). However, after 28 days of treatment, less CFU counts were measured in the lungs and spleens of the C10-treated mice than in sham-treated mice (p = 0.01 for both lungs and spleens CFUs, Friedman test). This decrease in bacterial burden was associated with a concomitant increase in body weight (Fig. 2, D). These data further indicated that PDE-I administration confers a therapeutic benefit in the tuberculous mouse.

### Evaluation of PDE-I administration in a granulomatous mouse model of TB

**Figure 3:**
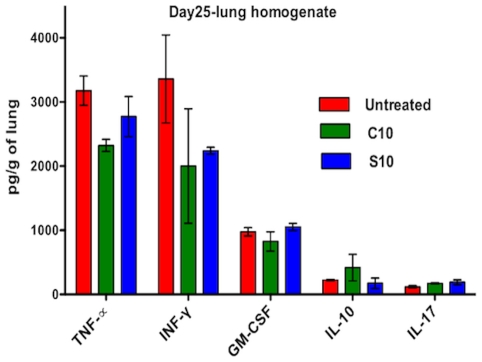
Cytokines profile of PDE-I monotherapy of C3HeB/FeJ infected mice. The administration of cilostazol and sildenafil decreases proinflammatory cytokine levels in the lungs of *M.tb.*-infected mice. C3HeB/FeJ mice infected with 3.67 log_10_CFU *M.tb.* were treated daily by oral gavage (starting the day after infection) with 10 mg/kg cilostazol (C10) or sildenafil (S10). At 25 days post-infection, lungs were homogenized, and the concentrations of TNF-α, IFG-γ, GM-CSF, IL-10 and IL17 were determined.

C3HeB/FeJ mice develop well-defined lung granulomas with central caseous necrosis in response to *M.tb.* infection [12]. Because the immune response in this model system generates a granulomatous disease in the mouse, we utilized C3HeB/FeJ mice to analyze the immunomodulatory effects of cilostazol and sildenafil, with the rationale being that immunomodulatory changes would be more pronounced within this model system. Mice were exposed to *M.tb.* by aerosol with a day 1 implantation of 3.67 log_10_CFU, and treatment was initiated the day following infection. C10 and S10 were administered daily (5 days/week) to both infected and uninfected C3HeB/FeJ mice. In this model system, statistically significant differences in lung CFU counts were not observed at day 3, 10, or 25 post-infection (Figure S1). However, administration of C10 and S10 resulted in decreased levels of tumor necrosis factor α (TNF-α) and interferon γ (IFN-γ) and increased levels of interleukin- (IL-)10 and IL-17 in mouse lung homogenates, relative to the levels in infected, untreated mice (Fig. 3). Uninfected mice treated with C10 or S10 did not exhibit altered cytokine profiles relative to uninfected, untreated mice (data not shown). Thus, these data suggest that cilostazol and sildenafil can modulate at least the Th1 balance in mouse granulomatous TB.

### Addition of PDE-Is to the standard TB chemotherapy regimen

**Figure 4:**
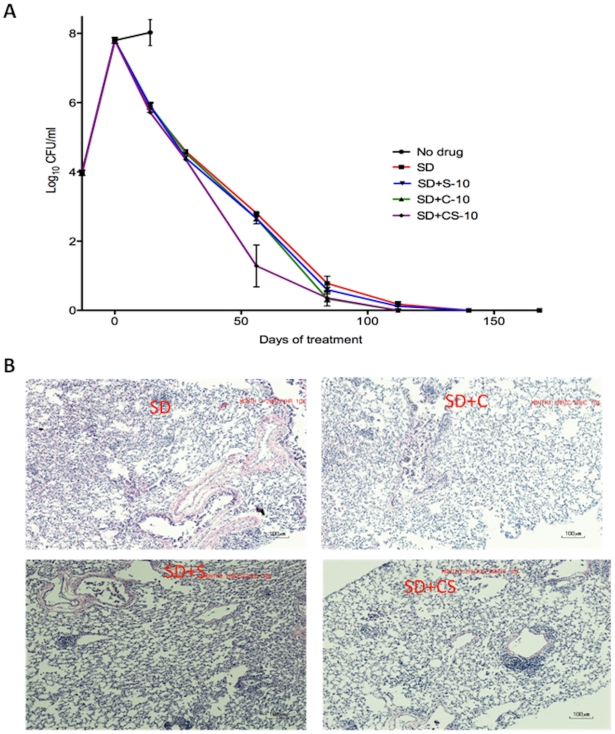
Addition of PDE-Is to standard TB therapy. The addition of PDE-Is to standard TB therapy reduces bacterial load, lung pathology and time to lung sterilization, and does not negatively interact with rifampin in *M.tb.*-infected mice. BALB/c mice were infected with 3.97 log_10_CFU *M.tb* and treatment was started 14 days post-infection. The mice were treated daily by oral gavage with standard therapy (SD, which is 2 months 10 mg/kg rifampin (R), 25 mg/kg isoniazid H), and 150 mg/kg pyrazinamide (Z), followed by 4 months of R and H), SD plus 10 mg/kg sildenafil (S10), cilostazol (C10) or both (CS10). (**A**) CFU counts in mouse lungs. (**B**) Histopathology analyses of mouse lungs (hematoxylin and eosin stained lung sections).

Our analyses of cilostazol and sildenafil indicated that these PDE-Is do not harm the tuberculous mouse, and in fact may provide a benefit to infected mice in our model systems. Thus, these data generated from PDE-I monotherapy experiments prompted us to analyze the effect of cilostazol and sildenafil when used as adjunctive therapy to the existing TB standard treatment regimen. We analyzed the bactericidal and sterilizing value of the addition of C10, S10 and C10+S10 to the standard 6 months short-course drug regimen, which is 2 months of treatment with rifampin (R), isoniazid (H) and pyrazinamide (Z), followed by 4 months of treatment with R and H. For this experiment, BALB/c mice were infected via aerosol with an implantation of 3.97 log_10_CFU (determined day 1 post-infection) (Fig. 4, A). Administration of drugs was initiated 14 days post-infection, when the lung bacterial load had reached 7.8 log_10_CFU. All untreated mice became moribund within 21 days of infection, and the mice, which survived to day 28 post-infection, had an average of 8.02 log_10_CFU in their lungs. At 28 days of treatment, mice with regimens containing adjunctive cilostazol (C10 or C10+S10) began to exhibit decreased bacterial lung burden relative to mice receiving the standard therapy alone (Fig. 4, A). Furthermore, the cilostazol-containing regimens decreased the time to lung sterilization by nearly 1 month, compared to mice receiving standard therapy; at 112 days of treatment, no CFUs were demonstrated from the lungs of mice receiving the standard therapy plus C10 or C10+S10, while the lungs of mice receiving standard (SD) therapy alone or with S10 had bacterial loads of 0.18 and 0.12 log_10_CFU, respectively. This decrease in bacillary load in the mice receiving cilostazol was associated with fewer lung lesions (18 for SD alone, 10 for SD+C10, 13 for SD+S10 and 14 for SD+S10+C10, P = 0.49) at day 112 of treatment (Fig. 4, B). Hence, these data indicate that the addition of C10 or C10 plus S10 as adjunctive drugs to the current TB treatment regimen could reduce the duration of treatment necessary to achieve bacterial clearance.

To analyze the effect of the PDE-Is on relapse following completion of treatment, ten mice from each group were maintained for an additional 4 months following termination of chemotherapy. At the end of this 4 months period, the lungs from all of the mice treated with standard therapy remained culture-negative, as did the lungs from all mice treated with adjunctive PDE-Is (Table 1). These data indicate that, in addition to decreasing the time to sterilization, incorporation of C10 into the standard regimen did not increase the likelihood of relapse during the four months following the end of therapy.

**Table 1:** Proportion of mice with no detectable CFUs during TB standard therapy with and without PDE-I.

Days of treatment	SD	SD+S	SD+C	SD+CS
**56**	0/5	0/5	0/5	2/5
**84**	1/5	0/5	3/5	1/5
**112**	2/5	3/5	5/5	5/5
**140**	5/5	5/5	5/5	5/5
**168**	5/5	5/5	5/5	5/5
**168+112**	10/10	10/10	10/10	10/10

The proportions of mice with no detectable CFUs were determined by homogenizing and plating the entire lungs from 56 to 168 days of treatment. Treatment was terminated after 168 days, and groups of mice were kept for an additional 4 months (168+112). Shading indicates lung sterilization in all mice in the group. SD: standard drug therapy (see Fig. 4 legend for details); S: 10 mg/kg sildenafil; C: 10 mg/kg cilostazol; CS: 10 mg/kg each of sildenafil and cilostazol.

### Cilostazol does not reduce the efficacy of rifampin

**Figure 5:**
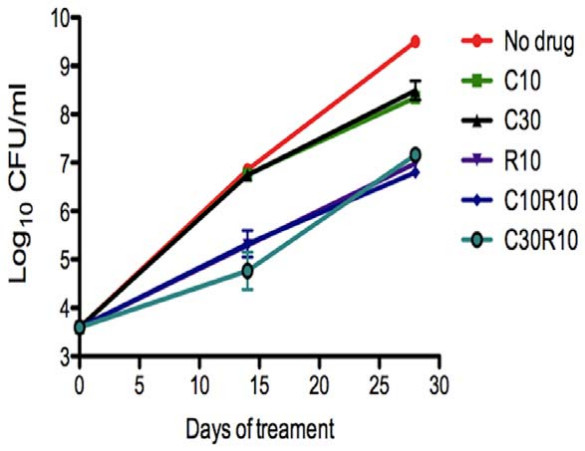
Interaction of PDE-Is with Rifampin. BALB/c mice were infected with 3.6 log_10_CFU *M.tb.* and were treated daily by oral gavage (starting the day after infection) with 10 or 30 mg/kg cilostazol (C10 and C30, respectively), 10 mg/kg rifampin (R10), C10 plus R10, or C30 plus R10. Lung CFU counts were determined on days 14 and 28 post-infection.

One complication in the development of new TB drug regimens is that many compounds have negative pharmacokinetic interactions with the first-line drug rifampin. Although our data do not suggest or support that a drug-drug interaction between cilostazol and rifampin (R) occurs, we felt it would be prudent to test for this specifically. BALB/c mice were infected by aerosol with 3.6 log_10_CFU. On the day following infection, mice were treated with either C10, C30, R10, C10+R10 or C30+R10. The addition of cilostazol at either concentration to rifampin did not impact rifampin activity after 14 and 28 days of treatment (Fig. 5).

Taken together, our data indicate that cilostazol can safely be added to the standard short-course TB chemotherapy regimen, and that the addition of cilostazol decreases the duration of treatment necessary to achieve bacterial clearance in the lungs.

## Discussion

In this communication, we have presented data suggesting that the PDE3-I cilostazol, an FDA-approved drug used for treatment of claudication, should be investigated for use as a treatment-shortening adjunctive drug to the standard TB short-course chemotherapy regimen. Using two different mouse models of TB, we have shown that administration of cilostazol provides a therapeutic effect for tuberculous mice when administered as monotherapy (Figs. 2, 3). Importantly, we have shown that addition of cilostazol to the standard 6 months short-course TB drug regimen resulted in sterilization of the mouse lungs one month earlier than standard treatment alone (Fig. 4, Table 1).

The duration of current TB drug regimens is widely acknowledged as contributing to treatment failures, and such failures are associated with the development of bacterial drug resistance and continued transmission of *M.tb.* [13]–[15]. Hence, shortening the length of treatment has the potential to improve treatment adherence and bacterial clearance, while reducing *M.tb.* transmission and the development of drug resistant organisms. Thus, the treatment-shortening potential of cilostazol could significantly contribute to TB control worldwide.

The use of adjuvant immunomodulatory therapy for the treatment of TB has been considered for more than a decade. In 2007, the WHO published a report on immunotherapeutic interventions for TB and concluded that adjunctive immunotherapy, in addition to standard chemotherapy, has the potential to shorten TB treatment duration, thereby improving the outcome of TB patients [16]. The conclusions of this report resulted in a WHO recommendation to fast track studies of immunomodulatory drugs in TB treatment. However, the few studies of adjuvant immunotherapy for TB that have been described report mixed outcomes, with multiple side effects associated with long-term use [17]. In contrast to immunomodulatory treatments such as prednisolone, thalidomide analogs and etanercept, cilostazol is well-tolerated by the host and is safe for long-term use in patients [18], [19]. The roles of TNF-α and the T cell response in TB have been largely debated. Immunomodulatory agents that inhibit TNF-α have been associated with both positive and adverse outcomes in TB patients [17], [20], [21]. While this topic remains controversial, it is becoming increasingly evident that it is the balance of TNF-α and other cytokines that influences the outcome of TB [22]. Our data indicate that the addition of cilostazol to the standard TB treatment regimen reduces TNF-α levels in mouse lungs, compared to untreated mice (Fig. 3), and that this decrease in TNF-α is not associated with any adverse effects on disease parameters within mice. Hence, our data suggest that the addition of cilostazol to standard TB chemotherapy can modulate immune signaling to enhance the host's ability to fight *M.tb.* infection.

Our finding that administration of cilostazol leads to decreased TNF-α levels is in agreement with results from several mechanistic studies of cilostazol. Hattori and colleagues have demonstrated that cilostazol inhibits TNF-α-induced nuclear factor-κB (NF-κB) activation in a dose dependent manner; this group also reported that cilostazol attenuated the TNF-α-induced gene expression of proinflammatory and cell adhesion molecules within THP-1 human monocytic cells [23]. Similar findings have been reported for PDE3-I-induced inhibition of TNF-α and NF-κB-dependent gene expression in rat vascular smooth muscle cells [24], [25]. Mendes and colleagues have shown that cilostazol administration dose-dependently decreases TNF-α levels in a mouse model of intraperitoneal adhesion [26]. Thus, the findings of these researchers support our data and suggest a possible TNF-α/NF-κB mechanism of cilostazol action in our tuberculosis model systems.

Sildenafil and Cilostazol have shown divergence of mechanism. Sildenafil (S10) prolonged the time to death of mice, decreased the level of TNF-α but did not impact the CFUs both in either mono- or multidrug regimens. This divergence may be explained by the differences in the mechanisms of action and the target of PDEs. PDE5 exhibits high affinity for cGMP while PDE4 specifically hydrolyses cAMP and PDE3 both cAMP and cGMP [27]. PDE3 is prominent in macrophages, endothelial cells, platelets and airway smooth muscle cells, while PDE5 is expressed in pulmonary vascular smooth muscle of pulmonary arteries and veins, bronchial blood vessels and airway smooth muscle. PDE4 is expressed more in the neutrophils, eosinophils, macrophages and endothelial cells [9].

This study began with an *in vitro* screening of PDE-Is in *M.tb.*-infected THP-1 cells using the laboratory/cell biology inhibitors trequinsin (PDE3-I) and MBM (PDE5-I). Addition of these chemicals to cell culture media resulted in decreased intracellular cAMP levels in the infected cells (Fig. 1), thus indicating that the host-pathogen infection balance could be altered in the presence of these PDE-Is. While further cell-based characterization of this observation will certainly reveal critical cell biology effects of these inhibitors, our interest was to examine their *in vivo* potential to influence the host-pathogen relationship. Thus, we shifted our studies directly into mouse experiments (rather than first stepping up to a primary cell culture model), as we felt this was a more representative model system to in which to examine the influence of PDE-Is on the host-pathogen relationship. Because FDA-approved PDE3-I (cilostazol) and PDE5-I (sildenafil) drugs were available, we utilized these compounds four our *in vivo* experiments, with the rationale being that these same compounds could be tested in humans, should the data suggest a positive effect and a lack of drug-drug interactions. However, the effects of cilostazol and sildenafil in the cell culture model have yet to be examined.

The PDE-I activity of cilostazol and sildenafil is accompanied by vasodilation, and hence it is possible that this latter activity could be important in the sterilizing role of these drugs in TB, as it has been demonstrated that microthrombi develop around mycobacterial lung lesions in a rabbit model of TB [27]. While our study design did not specifically address the role of vasodilation in TB treatment outcome, our data do not suggest that this is the mechanism of action of cilostazol, as treatment with sildenafil, which also has vasodilatory properties, was not associated with a decreased time to lung sterilization in infected mice. Thus, our data indicate that it is the specific PDE3-I activity of cilostazol that contributes to enhancement of standard TB therapy.

Recently, Koo and colleagues reported that use of the PDE4-I thalidomide analog CC-3052 (administered by oral gavage) in combination with isoniazid (delivered *ad libitum* in drinking water) resulted in a significant decrease in lung CFU counts and lung pathology in a mouse model of TB; they further demonstrated that administration of CC-3052 was associated with decreased *tnf-a* gene expression [28]. Subsequently, this group also reported similar findings in a rabbit pulmonary TB model [29], [30]. These exciting findings support the concept that PDE-Is can influence the host response to *M.tb.* While we did not examine inhibition of PDE4-Is in this study, we have shown that the addition of a PDE3-I and a PDE5-I to the standard 6-month TB chemotherapy regimen decreased the time to bacterial clearance in the lungs of infected mice. Thus, similar patterns were observed using type III, IV and V PDE-Is, and our data suggest that an FDA-approved PDE4-I could also be examined in a full chemotherapy model of TB.

In summary, we have demonstrated that the addition of the PDE3-I cilostazol to the standard TB chemotherapy regimen leads to lung sterilization one month earlier than treatment with standard therapy alone in a murine model of TB. This decreased time to bacterial clearance was associated with improved health measures, both on gross and histopathologic disease parameters. Shortening the duration of TB treatment is widely acknowledged as a mechanism for improving TB control and limiting the development of drug resistant *M.tb.* [13]–[15]. Cilostazol is currently an FDA-approved drug for the treatment of intermittent claudication and is safe for prolonged use in humans, as opposed to PDE4-Is, which are known to be associated with side effects [31]. Hence, cilostazol represents an existing available drug, which may be repurposed as an adjunctive agent for the treatment of TB.

## Materials and Methods

### Ethics Statement

This study was carried out in strict accordance with the recommendations in the Guide for the Care and Use of Laboratory Animals of the National Institutes of Health. All procedures described in this communication have been approved by the Johns Hopkins University Animal Care and Use Committee.

### Animals

Approximately 6 week-old female mice were used for all experiments. BALB/c mice were obtained from Charles River Laboratories (Wilmington, MA, USA), and C3HeB/FeJ mice were purchased from The Jackson Laboratory (Bar Harbor, ME, USA). Mice were maintained in an animal biosafety level 3 laboratory at all times.

### Bacterial and cell culture stocks

*Mycobacterium tuberculosis* strains H37Rv and CDC1551were obtained from the Johns Hopkins Center for Tuberculosis Research stocks. For preparation for infections, the mycobacteria were grown in 7H9 Middlebrook liquid medium supplemented with oleic acid-albumin-dextrose-catalase, 0.5% glycerol and 0.05% Tween-80. THP-1 cells were obtained from ATCC (TIB-202™), and were grown in RPMI medium supplemented with L-glutamine and fetal bovine serum.

### Aerosol infection procedure

Aerosol infections were performed with bacterial cultures diluted appropriately to achieve the desired inoculum using the Glas-col Inhalation Exposure System, per the manufacturer's instructions. For every infection, 3–5 mice were sacrificed the following day to determine the day 1 lung implantation. Lungs were dissected and homogenized in phosphate buffered saline (PBS). The lung homogenates were then serially diluted and plated in duplicate on selective 7H11 agar plates (Becton-Dickinson); plates were incubated for 3 weeks at 37°C and colonies were counted.

### Drug preparation and administration

Trequinsin and MBM were obtained from EMD Millipore Bioscience. Cilostazol (trade name Pletal®) is manufactured by Otsuka Pharmaceutical Co., and Sildenafil (trade name Viagra®) is manufactured by Pfizer. Isoniazid and rifampin were obtained from Sigma-Aldrich, and pyrazinamide was obtained from Fisher Scientific. Stock solutions were prepared weekly using distilled water or PBS and stored at 4°C.

All drugs were administered to mice by oral gavage. Drug solutions were prepared such that the desired concentration would be delivered in a 0.2 ml total volume. Rifampin was given 1 hour after administration of other drugs to avoid an adverse pharmacokinetic interaction [32].

### Measurement of intracellular cAMP in THP-1 cells

THP-1 cells were plated at 5×10^5^ per well in a 6-well cell culture dish (1 ml/well) and were stimulated with 50 ng/ml of phorbol 12-myristate 13-acetate; cells were then incubated overnight at 37°C/5% CO_2_ to allow for cell attachment. Trequinsin and MBM were administered at a concentration of 100 µM, and the cells were incubated with the drugs for 2 hours. Diluted mid-log phase *M.tb.* strain H37Rv was then added to the cells to achieve a multiplicity of infection of 1∶4. The bacterial suspension was incubated with the cells at 37°C/5% CO_2_ for 3 hours, after which the cells were washed and lysed with a digitonin/Tween-80 lysis buffer. The cAMP concentration in the cell lysates was determined using the Cyclic AMP Enzyme Immunoassay Kit (Assay Designs).

### Analyses of PDE-I treatment of BALB/c mice

All BALB/c mice were infected by aerosol as described above, with inocula as detailed in the main text. Treatment regimens were administered by oral gavage as described above and with doses as indicated in the main text. At the appropriate time points, five mice per group were sacrificed using isoflurane vapor. Total body weight was recorded, and then lungs were dissected and weighed. The lungs were kept in PBS for 24 hours at 4°C, and then examined for gross pathology. Lung samples were preserved in 10% formalin fixative for histopathology analyses, and the remaining lung was homogenized in PBS, serially diluted and plated on selective 7H11 agar plates. CFUs were counted after 3 weeks of incubation at 37°C.

### Analyses of PDE-I treatment of C3HeB/FeJ mice

C3HeB/FeJ mice were either not infected or infected with 3.67 log_10_CFU by aerosol as described above. Drug treatment was initiated the day after infection and was administered as described above. At the indicated time points, five mice per group were sacrificed using isoflurane vapor. Total body weight was recorded, and the lungs were dissected and weighed. Immediately following dissection, the lungs were homogenized in PBS, and then aliquotted for CFU determination (performed as described above for BALB/c mice) and cytokine measurement. Lung homogenates were stored at −80°C until the experiment was completed, and then all samples were analyzed in duplicate for cytokine levels using the Milliplex map mouse cytokine/chemokine kit (Millipore), and the samples were run on a Bio-plex system (Bio-Rad).

### Data Analysis

Lung or spleen CFU counts (x) were log-transformed as log_10_ (x+1) before analysis (means and standard deviations). The Student t-test, Bonferroni's Multiple Comparison Test or Kaplan Meier test were used. A p-value of 0.05 was considered significant for all statistical analyses.

## Supporting Information

Figure S1 **Log_10_ mean CFU evolutions of C3HeB/FeJ mice.** C3HeB/FeJ mice uninfected or aerosol infected with 3.67 log_10_ CFUs of *M. tuberculosis CDC1551* on Day1 were treated daily (5/7 days/week) with 10 mg/kg of Cilostazol or Sildenafil (5 mice per group and per time point: D-3, D-10 and D-25). The CFUs evolution is shown for infected mice during treatment.
(TIFF)
