# Supplementary figures and images for: Successful Shortening of Tuberculosis Treatment Using Adjuvant Host-Directed Therapy with FDA-Approved Phosphodiesterase Inhibitors in the Mouse Model
Source: PLoS One. 2012 Feb 3;7(2):e30749. doi: 10.1371/journal.pone.0030749 (PMC3272040; doi:10.1371/journal.pone.0030749)

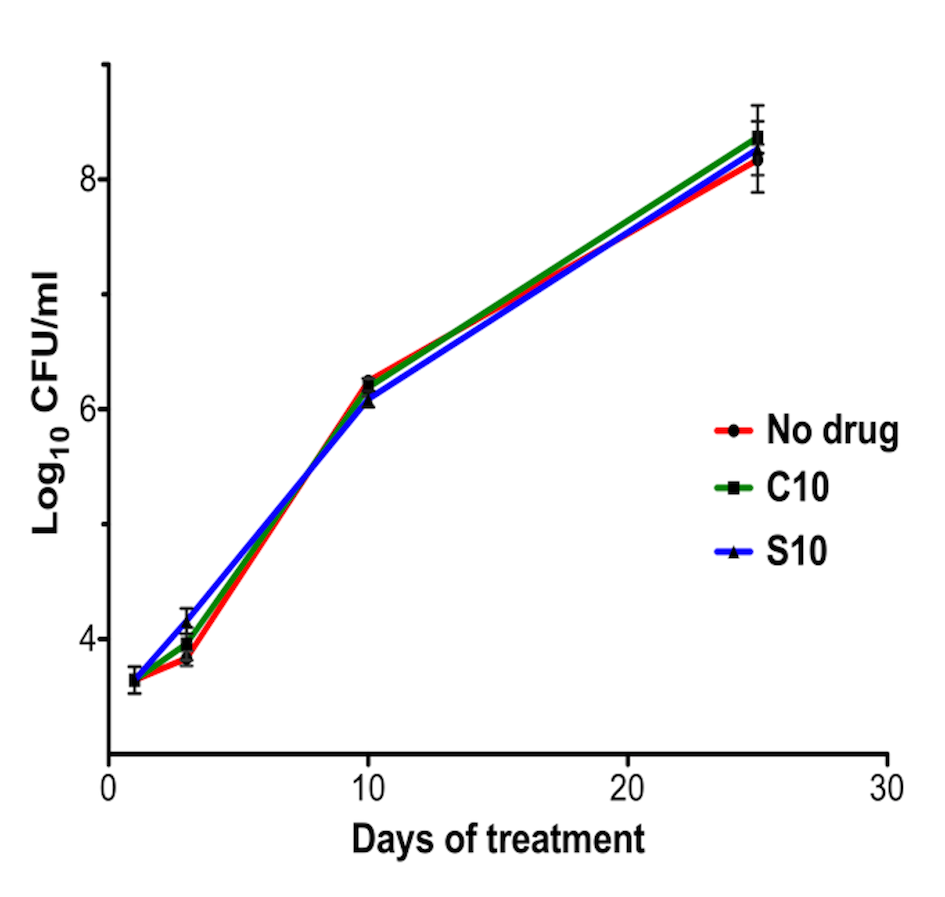

Supplement: Figure S1 — Log10 mean CFU evolutions of C3HeB/FeJ mice. C3HeB/FeJ mice uninfected or aerosol infected with 3.67 log10 CFUs of M. tuberculosis CDC1551 on Day1 were treated daily (5/7 days/week) with 10 mg/kg of Cilostazol or Sildenafil (5 mice per group and per time point: D-3, D-10 and D-25). The CFUs evolution is shown for infected mice during treatment. (TIFF) [file pone.0030749.s001.tiff]
